# Golgi Dynamics: The Morphology of the Mammalian Golgi Apparatus in Health and Disease

**DOI:** 10.3389/fcell.2019.00112

**Published:** 2019-07-03

**Authors:** Christian Makhoul, Prajakta Gosavi, Paul A. Gleeson

**Affiliations:** The Department of Biochemistry and Molecular Biology, Bio21 Molecular Science and Biotechnology Institute, The University of Melbourne, Melbourne, VIC, Australia

**Keywords:** Golgi ribbon, Golgi morphology, cell sensing, Golgi stacks, signaling

## Abstract

In vertebrate cells the Golgi consists of individual stacks fused together into a compact ribbon structure. The function of the ribbon structure of the Golgi has only begun to be appreciated (De Matteis et al., 2008; Gosavi and Gleeson, 2017; Wei and Seemann, 2017). Recent advances have identified a role for the Golgi in the regulation of a broad range of cellular processes and of particular interest is that the modulation of the Golgi ribbon is associated with regulation of a number of signaling pathways (Makhoul et al., 2018). Various cell responses, such as inflammation, and various disorders and diseases, including neurodegeneration and cancer, are associated with the loss of the Golgi ribbon and the appearance of a dispersed or semi-dispersed Golgi. Often the dispersed Golgi is referred to as a “fragmented” morphology. However, the description of a dispersed Golgi ribbon as “fragmented” is inadequate as it does not accurately define the morphological state of the Golgi. This issue is particularly relevant as there are an increasing number of reports describing Golgi fragmentation under physiological and pathological conditions. Knowledge of the precise Golgi architecture is relevant to an appreciation of the functional status of the Golgi apparatus and the underlying molecular mechanism for the contribution of the Golgi to different cellular processes. Here we propose a classification to define the various morphological states of the non-ribbon architecture of the Golgi in mammalian cells as a guide to more precisely define the relationship between the morphological and functional status of this organelle.

## Background

The basic unit of the Golgi apparatus is usually considered to be a stack of cisternae which is highly polarized, with the *cis*-face receiving cargo from the ER and the *trans*-face of the stack, the TGN, associated with sorting cargo for post-Golgi export (Boncompain and Perez, 2013). However, the organization of these mini-stacks in the cell varies amongst different organisms. In plants and invertebrates individual Golgi stacks are scattered independently throughout the cytoplasm whereas in most vertebrate cells during interphase, individual Golgi stacks are fused together into a compact ribbon structure located in close proximity to the MTOC (Wei and Seemann, 2017). The structure of the Golgi ribbon in mammalian cells is best revealed by electron microscopy (Rambourg and Clermont, 1997); high resolution optical microscopy using *cis* and *trans* markers can also detect the ribbon organization (Gosavi et al., 2018). An important question under investigation in the field is the relevance of the Golgi ribbon structure and the functional differences of the Golgi ribbon compared with “isolated Golgi mini-stacks” or other states of Golgi architecture. In other words what is the evolutionary advantage of the more complex ribbon morphology of the Golgi apparatus in vertebrate cells and what functions may be regulated by a transition to a non-ribbon structure?

Studies over the past few years are revealing that Golgi membranes provide a platform for the regulation of a range of cellular processes including cell polarization (Kupfer et al., 1983), directed migration (Millarte and Farhan, 2012), stress (Sasaki and Yoshida, 2015), DNA repair (Farber-Katz et al., 2014), mitosis (Rabouille and Kondylis, 2007), metabolism (Abdel Rahman et al., 2015), pro-inflammatory responses (Chen and Chen, 2018) and autophagy (Yamamoto et al., 2012; Lamb et al., 2013). Indeed, in mammalian cells there is now considerable evidence that the Golgi, like other intracellular organelles, can act as a cell sensor (Farhan and Rabouille, 2011; Mayinger, 2011; Millarte and Farhan, 2012; Sasaki and Yoshida, 2015; Luini and Parashuraman, 2016; Gosavi and Gleeson, 2017; Makhoul et al., 2018). It is also becoming clear that the precise morphology of the Golgi is relevant to the regulation of a number of these cell processes (Makhoul et al., 2018). The association of Golgi morphology with signaling was borne out of a genome wide kinome and phosphatome screen which identified a large cohort of kinases and phosphatase (20% of the total in the genome) that influenced the morphology of the Golgi (Chia et al., 2012). The changes in Golgi morphology included either fragmentation of the Golgi (loss of Golgi ribbon) or the formation of a very compact and condensed Golgi in the perinuclear location. The relevance of actin in the regulation of Golgi morphology was highlighted in this study by the identification of a number of kinases, for example ROCK1 and PAK1, which regulate actin dynamics and modulate Golgi structure (Chia et al., 2012). Other genome wide analyses have also highlighted the likelihood that the Golgi can receive and transmit a wide variety of signals that could influence, not only membrane transport pathways, but also other processes, apoptosis, mitosis, autophagy and stress responses (Farhan et al., 2010; Millarte et al., 2015).

The Golgi ribbon structure is highly dynamic and can undergo very rapid remodeling during a range of different conditions. For example, during mitosis the disassembly of the Golgi ribbon is an early event in G2/M transition and plays an important role as a cell cycle checkpoint in promoting mitotic entry (Wei and Seemann, 2010; Corda et al., 2012). The regulation of Golgi dynamics is mediated by interactions between molecular scaffolds located on the Golgi membrane and the cytoskeleton. MT dynamics can regulate the location of the Golgi ribbon at the centrosome and the repositioning of the Golgi to facilitate polarized trafficking and directed secretion (Millarte and Farhan, 2012; Sanders and Kaverina, 2015). In addition, membrane components of the Golgi can also nucleate and stabilize microtubules at both the *cis*- and *trans*-Golgi, and therefore the Golgi itself is also a MTOC (Efimov et al., 2007; Wu et al., 2016). Actin-mediated processes also contribute to the form and function of the Golgi and at least nine Golgi-localized molecular scaffolds have been identified which interact with the actin cytoskeleton [see review (Gosavi and Gleeson, 2017)]. Enhancement of actin polymerization at the Golgi results in dispersal of the ribbon, whereas inhibition of actin polymerization with specific drugs such as latrunculin A results in compaction of the Golgi (Lazaro-Dieguez et al., 2006; Makhoul et al., 2018, 2019). Given these regulatory networks that modulate the Golgi ribbon structure, we consider it very plausible that the balance between the Golgi ribbon and Golgi mini-stacks may define both the qualitative and quantitative responses of signaling pathways. The connection between Golgi morphology and signaling also has important ramifications on understanding the molecular basis of a number of diseases that are associated with the loss of the Golgi ribbon and the appearance of a dispersed Golgi. For example, the survival of some cancer cells has been shown to be associated with a dispersed Golgi which reduces the level of apoptosis (Farber-Katz et al., 2014; Petrosyan, 2015).

## What Does Golgi Fragmentation Mean?

**FIGURE 1:**
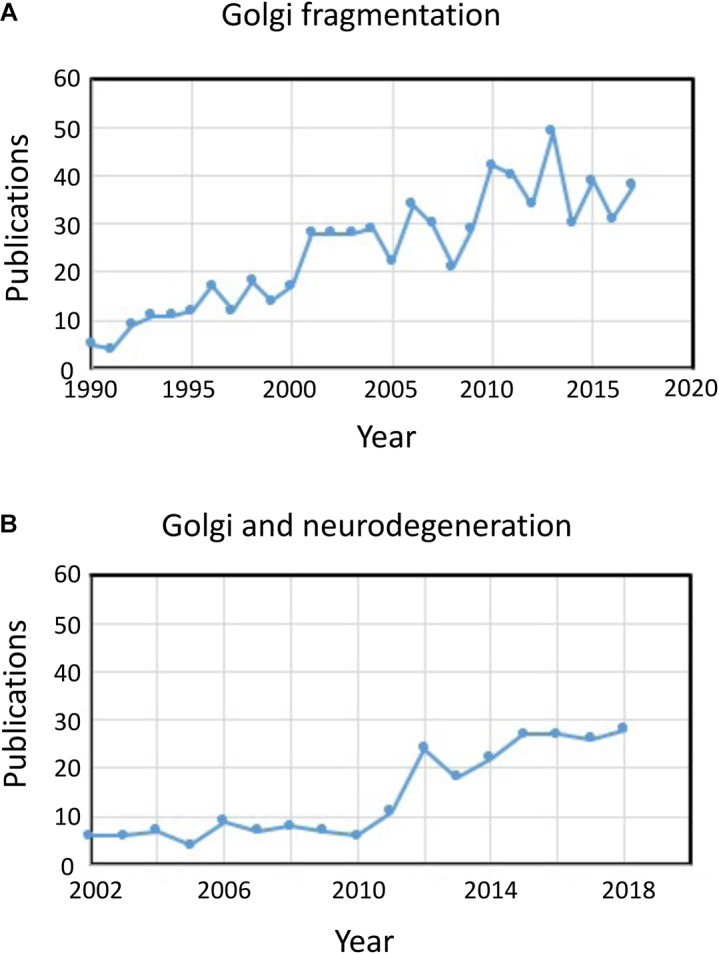
Publications identifying fragmentation of the Golgi ribbon. **(A)** Number of publications per year with the term Golgi fragmentation in either the title or abstract. **(B)** Number of publications per year where the Golgi has been examined in neurodegenerative diseases. Data is from Alexandru Dan Corlan. Medline trend: automated yearly statistics of PubMed results for any query, 2004. Web resource at URL: http://dan.corlan.net/medline-trend.html. Accessed: 2019-04-29.

The term “Golgi fragmentation” is commonly used to describe the morphological status of a dispersed Golgi in mammalian cells, as detected by optical microscopy stained with Golgi markers. A dispersed Golgi is often observed in experimental systems for example when cells are treated with drugs to perturb the cytoskeleton, e.g., nocodazole (Wei and Seemann, 2010), when membrane flux is perturbed, or when components of Golgi transport machinery, or the cytoskeletal interaction system, are knocked down, knocked out, or overexpressed (Zappa et al., 2018). In various physiological states, for example stress (Serebrenik et al., 2018) and pathological conditions, particularly cancer and neurodegeneration, the Golgi has often lost the typical compact juxtanuclear location and is observed by confocal microscopy as dispersed structures throughout the cytoplasm [see reviews by Gosavi and Gleeson (2017), Wei and Seemann (2017); in these pathological conditions the Golgi apparatus is also referred to as fragmented]. There are an increasing number of reports describing Golgi fragmentation under physiological and pathological conditions (Figure 1A). However, a problem with the use of the term “fragmented” is that it implies that the structural integrity of the Golgi is lost and that the morphology associated with the “fragmented” structure represents a disintegrated, abnormal or destroyed organelle. In a number of cases this is clearly misleading as the individual Golgi stacks may remain intact and can maintain the classical functions of the organelle, namely glycosylation and membrane transport. Indeed, dispersed Golgi mini-stacks occur in some specialized cells, such as differentiated myoblasts (Lu et al., 2001), differentiated neurons which contain individual Golgi stacks or “outposts” along dendrites (Lasiecka and Winckler, 2011), gastric parietal cells (Gunn et al., 2011), and uroepithelial cells of the urinary bladder (Kreft et al., 2010), without apparent deficiency in membrane transport and glycosylation. Rather, the dispersal of the Golgi ribbon associated with experimental and pathological conditions may reflect a shift of dynamic balance between the compact ribbon morphology and the individual Golgi mini-stacks or can result in perturbation of the ribbon architecture as well as the integrity of the Golgi stack. This is a relevant issue as the precise morphological status of the Golgi will very likely influence, in some cases the efficiency of transport and glycosylation (Puthenveedu et al., 2006), and in other cases a variety of signaling networks but not necessarily transport or glycosylation. It is important to differentiate between intact Golgi mini-stacks and loss of the integrity of the Golgi stacks in defining what is meant by a “fragmented” Golgi. Here we review the different Golgi morphologies that have been detected and characterized in experimental, physiological and pathological setting.

## Rethinking the Terminology of Golgi Morphological States

**FIGURE 2:**
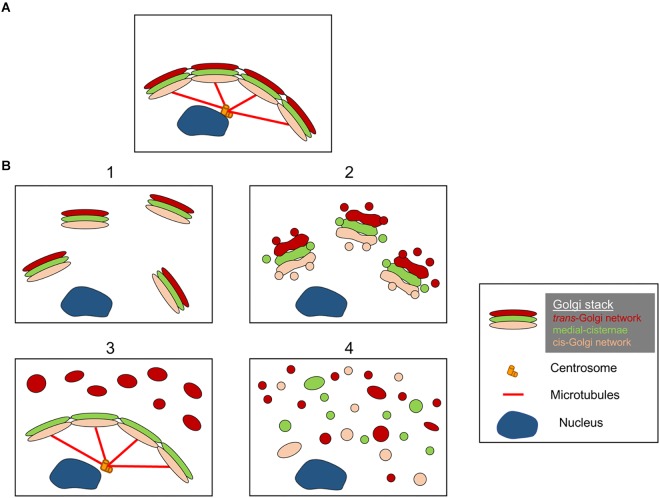
Model showing different Golgi morphologies following “fragmentation” of the Golgi ribbon structure. **(A)** Intact Golgi ribbon structure and **(B)** different scenarios showing loss of Golgi ribbon. (1) A scenario where intact Golgi mini-stacks are dispersed throughout the cytoplasm; (2) A scenario where the integrity of the dispersed Golgi stacks is compromised with shortened cisternae, swelling of cisternae and increase in Golgi associated tubules and vesicles; (3) A scenario where there is dispersal of one Golgi compartment. Here the TGN is selectively dispersed throughout the cytoplasm whereas the remainder of the stack remains in a ribbon structure; (4) Scenario where there is loss of ribbon and stacks with Golgi membranes dispersed predominantly as tubules and vesicles. Numbers refer to the classification of the Golgi morphologies given in text.

The structures of the Golgi fragments differ depending on the nature of pathway involved to perturb or modulate the Golgi ribbon. It is important to have a better ultrastructural characterisation of the Golgi “fragments” following loss of the Golgi ribbon as the functional outcome is likely to be very different depending on the precise Golgi structures. Aside from conditions that result in an elongated Golgi ribbon, we can identify from the literature at least 4 different scenarios associated with Golgi ribbon “fragmentation.” These are depicted in Figure 2 and described as follows:

(1) ***Conversion of Golgi ribbon to Golgi stacks***. Here the loss (or reduction of the length) of the Golgi ribbon is associated with dispersed, intact, Golgi mini-stacks. This situation occurs in a number of cell types and also in model systems, such as the treatment with nocodazole or the modulation of the levels of TGN golgin GCC88. Membrane transport appears to be largely unaffected under these conditions, with the exception of possibly large cargo (Ferraro et al., 2014; Lavieu et al., 2014), whereas mTOR signaling is reduced and autophagy enhanced (Gosavi et al., 2018).
(2) ***Loss of both Golgi ribbon and integrity of Golgi stacks***. In this scenario, the ribbon structure is lost and, in addition, cisternae of individual stacks are reduced in length and/or number and may also be associated with swollen compartments, for example the knockout of GM130 (Liu et al., 2017) and mutations of COG subunits (Blackburn and Lupashin, 2016). Membrane transport and glycosylation are likely to be affected in this scenario along with various signaling networks.
(3) ***Dispersal of one Golgi compartment***. In this scenario, only one Golgi compartment is dispersed, such as the dispersal of the TGN recently reported activation of the NLRP3 inflammasome (Chen and Chen, 2018). The remainder of the stack/ribbon remains unaffected. Identifying these Golgi structures requires incorporating EM and optical microscopy and a number of Golgi markers across the stack to define the precise changes in Golgi morphology.
(4) ***Conversion of Golgi ribbon to tubulovesicular elements***. In this scenario, both the Golgi ribbon and stacks are extensively perturbed with dramatic increases in tubulovesicular structures, as in the case during mitosis (Wei and Seemann, 2017), various drugs and treatment of cells with amyloid β (Joshi et al., 2014). Here one would anticipate an impact on many of the functions of the Golgi.

We do not infer that Figure 2 represents the only morphologies of Golgi “fragments” and it is possible that additional scenarios will be identified as the structures of Golgi “fragments” are investigated more extensively.

## Examples of a Relationship Between Golgi Morphology and Cell Process

The co-ordination of changes in Golgi morphology and various cell processes has received considerable attention. For a more detailed summary of the background information of the processes influenced by the changes in Golgi morphology, such as trafficking, glycosylation, stress, DNA repair, the reader is referred to a number of recent reviews (Farhan and Rabouille, 2011; Millarte and Farhan, 2012; Sasaki and Yoshida, 2015; Gosavi and Gleeson, 2017; Makhoul et al., 2019). Below are some examples to highlight the range of cell processes that are regulated or co-ordinated by different morphological states of the Golgi. In a number of other instances, such as some cancers and stress responses, the Golgi ribbon is dispersed as fragments, however, the morphology of these Golgi fragments has not been well characterized. The discussion herein will be focused on the examples where the Golgi morphology is well defined.

### DNA Repair and Cancer

There is an intimate association between Golgi morphology and the DNA damage response (Farber-Katz et al., 2014). The Golgi membrane tether, GOLPH3, is an oncogene and overexpression of GOLPH3 results in enhanced cell survival following DNA damage (Scott et al., 2009; Farber-Katz et al., 2014). Conversely, loss of GOLPH3 prevents the dispersion of the Golgi ribbon, enhances the Golgi ribbon and promotes apoptosis after DNA damage. The DNA damage response is mediated by the kinase DNA-PK, which phosphorylates GOLPH3 and promotes Golgi “fragmentation” by enhancing actin polymerization at Golgi membranes (Dippold et al., 2009). mTOR is modulated by the changes in Golgi morphology mediated by GOLPH3 (Scott et al., 2009) and is likely to contribute to the outcome of the DNA response. Hence, the precise Golgi structure is tied to cell survival and apoptosis. The identity of the Golgi structures following the dispersal of the Golgi ribbon by phospho-GOLPH3 remain to be characterized. Clearly future studies examining the relationship between Golgi fragmentation and mTOR signaling in cancer cells will be well worthwhile.

## Genetic Disorders Associated With Altered Golgi Morphology

Many diseases have been identified with monogenic disorders caused by inherited mutations of either components associated with transport machinery or of the enzymes resident in the Golgi. Many of these diseases are associated with fragmentation of the Golgi ribbon, for example, defects in the conserved oligomeric Golgi complex (COG) in congenital disorders of glycosylation (Miller and Ungar, 2012). In many cases the disorders are associated with pathologies restricted to a limited number of organs or tissues. The basis for tissue specificity is poorly understood, but likely due to deficiencies in glycosylation and secretion and also alterations in signaling networks associated with the loss of the Golgi ribbon, such as Golgi stress responses. The Golgi in all COG subunit knockout cell lines show moderate to severe change in morphology as characterized by EM, associated with loss of the ribbon, dilated cisternae and in some cases disruption of the mini-stacks. The application of EM was fundamental in defining the morphological changes (Blackburn and Lupashin, 2016).

### Neurodegenerative Diseases

The status of the Golgi in neurodegenerative diseases has recently received considerable attention (Figure 1B). Loss of the Golgi ribbon is a common feature of many neurodegenerative diseases including Alzheimer’s disease, Huntington disease, amyotrophic lateral sclerosis and Parkinson’s disease (Gonatas et al., 2006; Haase and Rabouille, 2015; Rabouille and Haase, 2015; Sundaramoorthy et al., 2015). It is very likely that the perturbations in the Golgi architecture in these diseases contributes to the pathological processes. In most cases the precise morphological structure of the Golgi fragments in these neurodegenerative diseases have not been defined. However, two experimental systems have recently investigated changes in Golgi structure and neuronal degeneration in some detail. Firstly, conditional knock out of GM130, a structural golgin which regulates the Golgi ribbon, in the central nervous system was demonstrated to cause Golgi fragmentation, atrophy of dendrites and neuronal degeneration in mice (Liu et al., 2017). EM analysis of the GM130 KO Purkinje cells showed a reduction of Golgi cisternal length and stacking and, in addition, the typical Golgi dendrite outposts were absenting in these GM130 -/- Purkinje cells (Liu et al., 2017). In a second study, hippocampal neurons from mice transgenic for the Swedish mutation of amyloid precursor protein (APP) and a mutant presenilin 1 subunit of γ-secretase, where both mutations are associated with early onset Alzheimer’s disease, revealed extensive Golgi fragmentation by optical microscopy (Joshi et al., 2014). Quantitative EM of neurons in these mice showed a reduction in the number and length of the cisternae in the stacks compared with neurons from wild type mice. In addition, cisternae were swollen. The changes in Golgi morphology in these primary neurons was shown to be directly associated with the elevated level of amyloid-β production. Primary neurons treated with synthetic amyloid-β also showed similar fragmentation of the Golgi as well as an increase in tubulovesicular structures associated with Golgi cisternae compared with untreated cells. The loss of the Golgi ribbon was due to phosphorylation of GRASP65, a Golgi structural protein which plays a key role in disassembly of the Golgi ribbon and stacks in mitosis (Joshi et al., 2014). Comparison of these two studies above is informative as the pathways mediating the changes in Golgi morphology differ in each case leading to differences in the morphology of the Golgi “fragments.” Consideration needs to be given as to how these different pathways affecting Golgi morphology influence the downstream responses.

### mTOR Signaling

Our lab has established an experimental approach to perturb the balance between the Golgi ribbon and Golgi mini-stacks by modulating the dose of GCC88, a golgin located at the TGN. This strategy allowed a stable cell line, HeLa-B6, to be established that lack a Golgi ribbon. We have shown that GCC88 regulates the balance between Golgi ribbons and mini-stacks by an actin dependent process (Makhoul et al., 2019) and identified intersectin-1 (ITSN1), a guanine nucleotide exchange factor for cdc42 (Hussain et al., 2001), as an interactor of GCC88 responsible for the loss of the Golgi ribbon (Makhoul et al., 2019). Analyses of HeLa B6 cells, which lack a Golgi ribbon, demonstrated reduced mTOR activity and an associated increase in autophagosome biogenesis (Gosavi et al., 2018). mTOR is one of the major signaling pathways of eukaryotic cells and known to be a negative regulator of autophagy (Wullschleger et al., 2006). Hence, the balance of Golgi stacks to Golgi ribbon has a direct effect on the mTORC1 pathway. The use of *cis*- and *trans*- compartment specific markers, and EM tomography, was critical in revealing the morphological changes in the Golgi ribbon.

### Inflammation

Inflammasomes of the innate immune system act as a scaffold for caspase 1-dependent activation of pro-inflammatory cytokines (Broz and Dixit, 2016). The NLRP3 (nucleotide-binding domain, leucine-rich-containing family, pyrin domain-containing-3) is a versatile inflammasome which can be activated by a range of microbial and non-microbial stimuli resulting in secretion of pro-inflammatory cytokines interleukin 1β (IL-1β) and interleukin 18 (IL-18) and programmed cell death by pyroptosis. A recent study has demonstrated that the activation of the cytosolic NLRP3 by stimuli involves recruitment of NLRP3 to dispersed TGN membranes to facilitate NLRP3 scaffold assembly (Chen and Chen, 2018). The dispersed TGN, but not the underlying other compartments, specifically recruits NLRP3, via PI4P, to assemble the downstream adaptor complex ASC which undergoes polymerization in the peri-nuclear region before recruiting caspase-1 to activate the downstream signaling pathway (Chen and Chen, 2018). Hence this important finding demonstrates that the modulation of the architecture of the TGN selectively, is critical in the activation of this pathway. The use of compartment specific markers (TGN and *cis*-Golgi) together with optical and EM analysis was critical in revealing the morphological changes in the Golgi ribbon.

## Concluding Remarks

The precise architecture of Golgi morphology is defined by the high resolution optical microscopy and by EM. The inclusion of this information in future studies in the field will provide a considerable insight into the dynamics of the Golgi, the pathways for perturbation of the ribbon structure and the functional consequences associated with these different pathways.

In summary, we propose that the use of the term “fragmented Golgi” is inadequate to describe Golgi structures associated with many treatments and conditions and the differences in these Golgi structures are likely to be relevant physiologically. Given the dynamic nature of the Golgi apparatus, it is possible that there may be a balance between different morphological states of the Golgi at any given time i.e., mini-stacks and ribbon structures. Understanding the dynamic balance between the different Golgi morphologies in molecular detail is critical for a full appreciation of this organelle during normal cell processes and also under pathological conditions. It will be fascinating to see what unfolds as we learn more about the cell sensing functions of this complex organelle and the relationship between these functions and Golgi structures.

## Data Availability

All datasets analyzed for this study are included in the manuscript and the supplementary files.

## Author Contributions

All authors listed have made a substantial, direct and intellectual contribution to the work, and approved it for publication.

## Conflict of Interest Statement

The authors declare that the research was conducted in the absence of any commercial or financial relationships that could be construed as a potential conflict of interest.

## Abbreviations

GM130: *cis*-Golgi matrix protein
GMAP210: Golgi microtubule associated protein
GOLPH3: Golgi phosphoprotein 3
GRASP: Golgi reassembly stacking protein
MTOC: microtubule organizing center
mTOR: mechanistic target of rapamycin
PI4P: phosphatidylinositol-4-phosphate
TGN: *trans*-Golgi network.
